# Fatigue after COVID-19 infection is associated with peripheral immunometabolic alterations affecting neuroimmune responses in the hippocampus

**DOI:** 10.1038/s41398-026-04250-9

**Published:** 2026-07-15

**Authors:** Nicole Mariani, Daniel Martins, Federico Imbeni, Madeline Kirkpatrick, Nuriza Tukiran, Silvia Alboni, Carmine Pariante, Alessandra Borsini

**Affiliations:** 1 Department of Psychological Medicine, IoPPN, King’s College London, London, UK; 2 Department of Neuroimaging, IoPPN, King’s College London, London, UK; 3 Department of Clinical Neurosciences and Mental Health, Faculty of Medicine, University of Porto, Porto, Portugal; 4 RISE-HEALTH (Neuroscience thematic line), Department of Clinical Neurosciences and Mental Health, Faculty of Medicine, University of Porto, Porto, Portugal; 5 Department of Life Sciences, University of Modena and Reggio Emilia, Via Campi, Modena, 287-41125 Italy

**Keywords:** Molecular neuroscience, Diagnostic markers

## Abstract

Fatigue is a common and disabling symptom reported following SARS-CoV-2 infection, yet the underlying biological mechanisms remain poorly understood. In this study, we investigated whether fatigue severity in individuals previously infected with SARS-CoV-2 is associated with immune and metabolic alterations in serum and whether these peripheral changes can influence hippocampal cell function in vitro. Serum cytokines, kynurenine pathway, and tryptophan-derived and monoamine-related metabolites were measured in a total of 38 individuals with past COVID-19 infection. Human hippocampal progenitor cells were exposed to 1% patient serum during proliferation and differentiation, with readouts including cytokine release, metabolite production, and markers of neurogenesis (doublecortin, DCX) and astrocytic reactivity (glial fibrillary acidic protein, GFAP; aquaporin-4, AQP4). Results show that fatigue severity correlates with lower serum levels of interleukin-8 (IL-8) and with lower levels of metabolites of the kynurenine pathway and tryptophan-derived and monoamine-related metabolites, including kynurenine (KYN) and quinolinic acid (QUIN), and 5-hydroxyindoleacetic acid (5HIAA). Exposure of hippocampal cells to serum from individuals with higher fatigue was associated with increased endogenous production of interleukin-13 (IL-13) and the kynurenine metabolite anthranilic acid (ANA) in the cell supernatant, as well as with increased neurogenesis (increased DCX expression) and enhanced astrocytic reactivity (increased GFAP expression). Notably, serum IL-8 level was inversely correlated with both cellular outcomes. Likewise, serum 5-HIAA levels were negatively correlated with IL-13 release, with mediation analysis indicating that 5-HIAA significantly mediated the association between fatigue severity and IL-13 production (71% explained). Overall, our results suggest that fatigue after COVID-19 infection is associated with neuroimmune and metabolic changes in hippocampal cells, involving peripheral serotonin metabolism (5-HIAA) and cytokine signalling (IL-13).

## Introduction

A substantial proportion of individuals experience persistent symptoms following acute SARS-CoV-2 infection, even after virological recovery. Among these, fatigue is one of the most frequently reported and debilitating complaints, often occurring independently of the severity of the initial infection, including in cases that did not require hospitalisation or intensive care [1, 2].

A growing body of evidence indicates that SARS-CoV-2 infection is followed by alterations in peripheral immune signalling that can extend beyond the acute phase of illness [3–7]. Increased circulating levels of pro-inflammatory cytokines, including interleukin (IL)-1β, IL-6, and tumour necrosis factor (TNF)-α, have been reported after infection and have been associated with fatigue and cognitive complaints [7, 8]. Longitudinal studies further suggest that, in some individuals, the resolution of acute inflammation is accompanied by changes in adaptive immune function, including features of T-cell dysfunction or immune attenuation [6, 7]. Such peripheral immune changes are thought to influence neuroimmune communication through established immune-to-brain signalling mechanisms, including cytokine-mediated effects on blood–brain barrier function and central inflammatory pathways [8, 9]. In line with this, neuropsychiatric research has highlighted that systemic inflammation associated with COVID-19 infection can impact central nervous system function, with proposed roles for immune–brain signalling, glial activation, and altered neuroinflammatory processes [10]. In parallel, immune activation during and after SARS-CoV-2 infection has been linked to changes in tryptophan metabolism and downstream kynurenine and monoamine-related pathways [11–13]. Pro-inflammatory cytokines are known to induce key enzymes of the kynurenine pathway, such as indoleamine 2,3-dioxygenase, kynurenine 3-monooxygenase, and kynureninase, thereby shifting tryptophan metabolism away from serotonin synthesis toward kynurenine-derived metabolites with neuroactive properties [14–19]. Together, these findings provide a mechanistic basis through which post-COVID-19 immune alterations may influence neuroimmune processes and brain plasticity, without assuming the presence of persistent post-acute symptoms. Despite growing evidence for immune and metabolic alterations following SARS-CoV-2 infection, experimental studies directly examining how post-infectious serum profiles influence human brain cells remain limited. In the present study, we investigated whether fatigue severity in individuals previously infected with SARS-CoV-2 is associated with persistent peripheral immune and metabolic alterations, and whether these serum changes can influence hippocampal cell function in vitro. We first analysed associations between fatigue severity and circulating cytokines, kynurenine pathway metabolites, and metabolites of tryptophan and metabolites related to key neurotransmitter systems (serotonin, dopamine, and glutamate) in serum samples from individuals with post-COVID-19 fatigue. We then exposed a validated human hippocampal progenitor cell model to direct treatment with the same serum samples to assess their effect on neurogenesis, astrocytic reactivity, and neuro-immune metabolic signalling. By combining patient serum analysis with cellular and molecular readouts, this study aimed to elucidate how post-COVID-19 immune and metabolic alterations may contribute to fatigue-related changes in hippocampal plasticity. We hypothesised that greater fatigue severity would be associated with altered peripheral cytokine and metabolite profiles, and that serum from individuals with higher fatigue would be associated with neuroimmune and metabolic changes in human hippocampal progenitor cells.

## Materials and methods

### Participants’ cohort

The study included serum samples from 38 individuals who had SARS-CoV-2 *mild* acute infection. Demographics of the cohort are summarised in Table 1. All participants were required to have had a biologically confirmed SARS-CoV-2 infection prior to enrolment. Confirmation was based on either a positive reverse-transcription polymerase chain reaction (RT-PCR) test from a nasopharyngeal swab or documented serological evidence of prior infection, in accordance with UK national testing guidelines at the time of acute illness. Participants with only clinically suspected but biologically unconfirmed COVID-19 were not included. Participants were recruited between 3 and 36 months after their initial infection, with 60.5 and 39.5% of the cohort sampled more than 12- and 24-months post-infection, respectively. Recruitment was conducted through King’s College Hospital NHS Foundation Trust and affiliated community networks. Eligible individuals were aged 16–65 years, fluent in English, and had the capacity to provide informed consent. Individuals were excluded if they met any of the following criteria: history of major neurological or psychiatric disorders, current substance misuse, systemic or central nervous system inflammatory conditions, chronic respiratory or cardiac disease, recent vaccination (within two weeks), pregnancy or breastfeeding, BMI > 30, or current use of immunomodulatory or psychoactive medications. Participants were pre-screened for symptom severity of fatigue using different scales: the global severity score of the Fatigue Assessment Inventory (FAI) for overall fatigue burden [20], the Multidimensional Fatigue Inventory (MFI) for physical, mental, and motivational dimensions [21], and the fatigue Visual Analogue Scale (VAS) providing a continuous measure of perceived fatigue intensity [22]. As fatigue is one of the most common symptoms after COVID-19 infection with a wide range of severity, and given that dichotomising continuous measures can obscure clinically relevant variation and reduce statistical power, all fatigue scales were analysed as continuous variables [23].

**Table 1:** Demographic and clinical characteristics of the cohort.

Characteristic	Value	*n*.
**Age (years)**		38
Mean ± SD	41.0 ± 10.3	
**Sex**		38
Female, n (%)	33 (86.8%)	
Male, n (%)	5 (13.2%)	
**BMI (kg/m²)**		38
Mean ± SD	23.9 ± 3.5	

The study received ethical approval from the UK Health Research Authority (IRAS ID: 308661) and was conducted in accordance with the Declaration of Helsinki and Good Clinical Practice (GCP) guidelines. All participants provided written informed consent prior to study procedures.

### Cell culture

Multipotent human hippocampal progenitor cell line HPC0A07/03 C (provided by ReNeuron, Surrey, UK) was used. This model has been previously validated using a hippocampal newborn neuron-specific marker, Prospero homeobox protein 1 (Prox1) [24]. Cells were left to proliferate in Dulbecco’s Modified Eagle Medium: Nutrient Mixture F-12 (DMEM/F-12) media supplemented with the growth factors (epidermal growth factor (EGF), basic fibroblast growth factor (bFGF) and 4-hydroxytamoxifen (4-OHT)). Differentiation was initiated by the removal of the growth factors and 4-OHT. Under standard differentiation conditions, this model generates a mixed neuronal and astrocytic population, consisting of approximately 70% neurons and 30% astrocytes, as previously reported. Detailed information on this cell line can be found in our previous publication [24].

### In vitro treatment with serum samples

Cells were plated on 96-well plates (Nunclon) at a density of 1.2 × 10^4^ cells per well. Following our established model [25], cells were plated simultaneously from the same passage to ensure consistency across experimental conditions, and serum samples from individual participants were applied to separate wells, each representing a distinct treatment condition. After 2 days of proliferation, cells were left to differentiate for 7 days. At this stage, cells were either fixed with 4% PFA for 20 min at room temperature and immunostained or cell supernatants were collected for cytokine measurements. During both proliferation and differentiation, cells were treated with media containing 1% serum (from each patient) and 0.5 mg/ml penicillin. The 1% serum concentration was selected based on our previously established human hippocampal progenitor cell model, where it allows assessment of patient-serum effects while preserving cell viability and differentiation capacity [25].

### Multiplex cytokine measurement in cell supernatant and serum samples

Cell supernatants and serum samples from individuals with past COVID-19 infection were used for cytokine measurement (IL-1β, IL-2, IL-4, IL-6, IL-8, IL-10, IL-12, IL-13, TNF-α, IFN-γ), using the Human ProInflammatory Multiplex Very-Sensitive Kit from Meso Scale Discovery (MSD) (Gaithersburg, MD) and the SECTOR Imager MSD device, according to the manufacturer’s instructions. We have excluded data of cytokines whose levels were below the lower limit of quantification (LLOQ) given by the company in 20% or more of the samples.

### Measurement of the kynurenine pathway and tryptophan-derived and monoamine-related metabolites

The concentrations of kynurenine pathway and tryptophan-derived and monoamine-related metabolites were measured in patient serum and in cell culture supernatants after 7 days of differentiation. Fifty µL of the sample were added with an equal volume of ice-cold 1 M perchloric acid (HClO_4_) fortified with a mix of the following stable isotope-labelled internal standard (final concentration 1 µM): L-kynurenine-d4, kunurenic acid-d5 (Buchem BV), and L-Tryptophan-d5 (Sigma-Aldrich, Burlington, MA, USA). Samples were centrifuged (15000 × g, 15 min), and the supernatants were collected and directly injected into liquid chromatography with tandem mass spectrometry (LC-MS/MS). Using an Agilent HP 1200 liquid chromatograph (Agilent, Milan, Italy), metabolites of the kynurenine pathway and tryptophan-derived and monoamine-related metabolites. Specifically, the following kynurenine pathway metabolites were measured: kynurenine (KYN), nicotinamide (NIC), 3-hydroxykynurenine (3HK), 3-hydroxyanthranilic acid (3HANA), kynurenic acid (KYNA), xanthurenic acid (XANA), anthranilic acid (ANA), and quinolinic acid (QUIN). Whereas the following tryptophan-derived and monoamine-related metabolites were measured: tryptophan (TRP), serotonin (5-hydroxytryptamine, 5HT), 5-hydroxyindoleacetic acid (5HIAA), N-acetylserotonin (A5HT), dopamine (DA), homovanillic acid (HVA) and glutamate (GLU). In the results section, we have reported only the metabolites that were detected. Chromatographic separations were carried out using a Discovery HS-F5 column (3-µm particle size, 150 × 2.1 mm, Supelco, Milan, Italy) using 0.1% formic acid in water and acetonitrile (ACN) as mobile phase. The high-performance liquid chromatography (HPLC) analyses were carried out using a linear elution profile of 15 min from 5 to 90% of ACN. The column was washed with 90% ACN for 3.5 min, then equilibrated for 5 min with 5% ACN. The flow rate was 0.5 mL/min. The injection volume was 20 µL. An Agilent 6410 triple quadrupole-mass spectrometer with an electrospray ion source operating in positive mode was used for detection. For cell culture supernatants, metabolite levels were interpreted relative to baseline culture media levels and therefore reflect net changes resulting from both cellular uptake and release.

### Immunocytochemistry (ICC)

Fixed cells were stained for markers of immature neurons (doublecortin, DCX; Alexa 488 donkey anti-rabbit; 1:1000, Invitrogen) and astrocytic reactivity (GFAP, AQP4). All cells were labelled using DAPI dye. For each marker (DCX, GFAP and AQP4), two parameters were quantified: mean fluorescence intensity and percentage of positive cells (calculated as the number of DAPI-labelled nuclei co-localising with the marker, divided by the total number of DAPI-labelled nuclei, expressed as a percentage).

### Statistical analysis

**Fig. 1:**
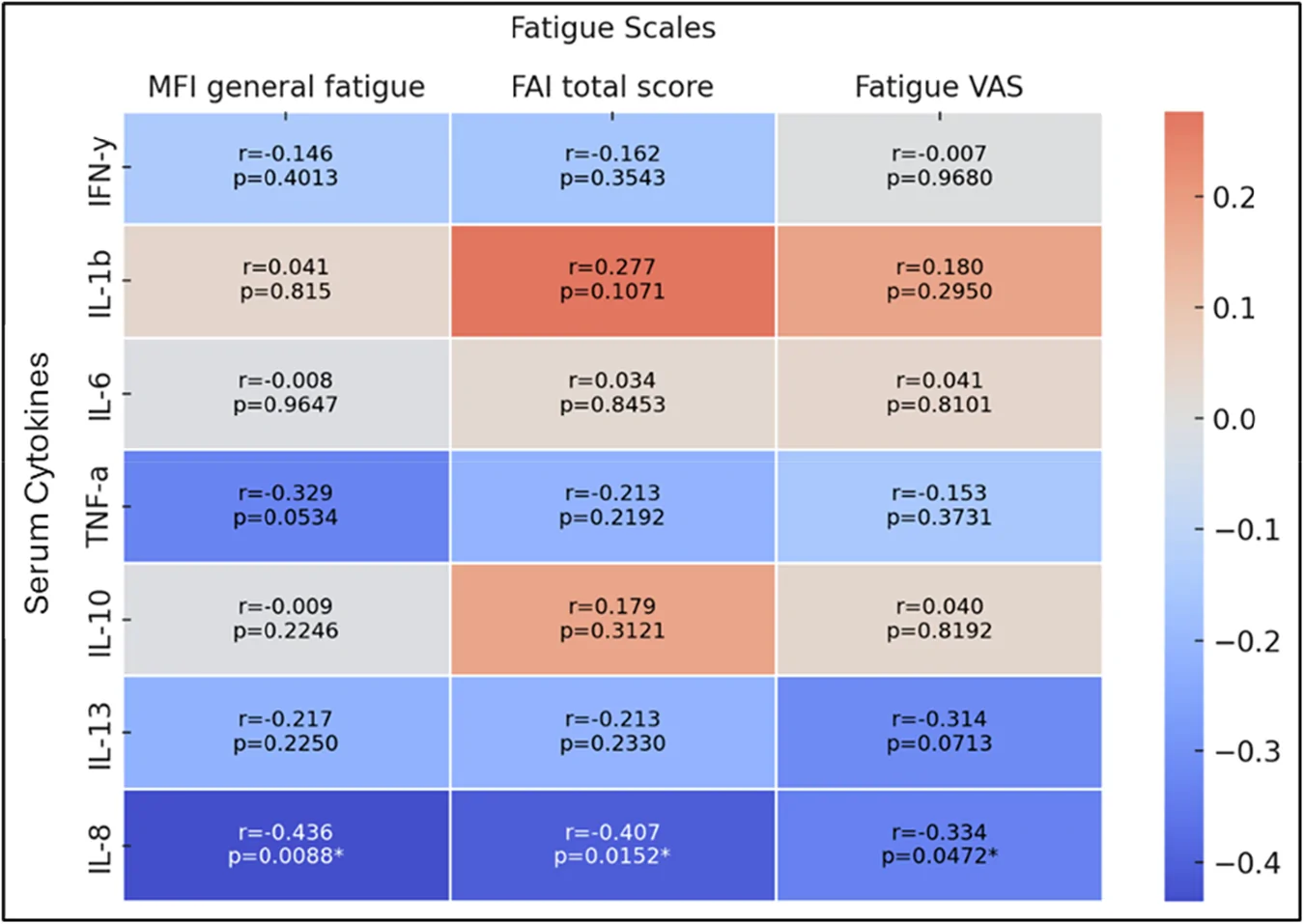
Heatmap showing the correlations between fatigue scales and serum cytokines. Correlation coefficients (*r*) are displayed inside each cell together with their corresponding *p*-values. The colour scale represents the strength and direction of the correlation, with red indicating positive and blue indicating negative associations. *p* < 0.05 was considered statistically significant and is indicated by an asterisk.

**Fig. 2:**
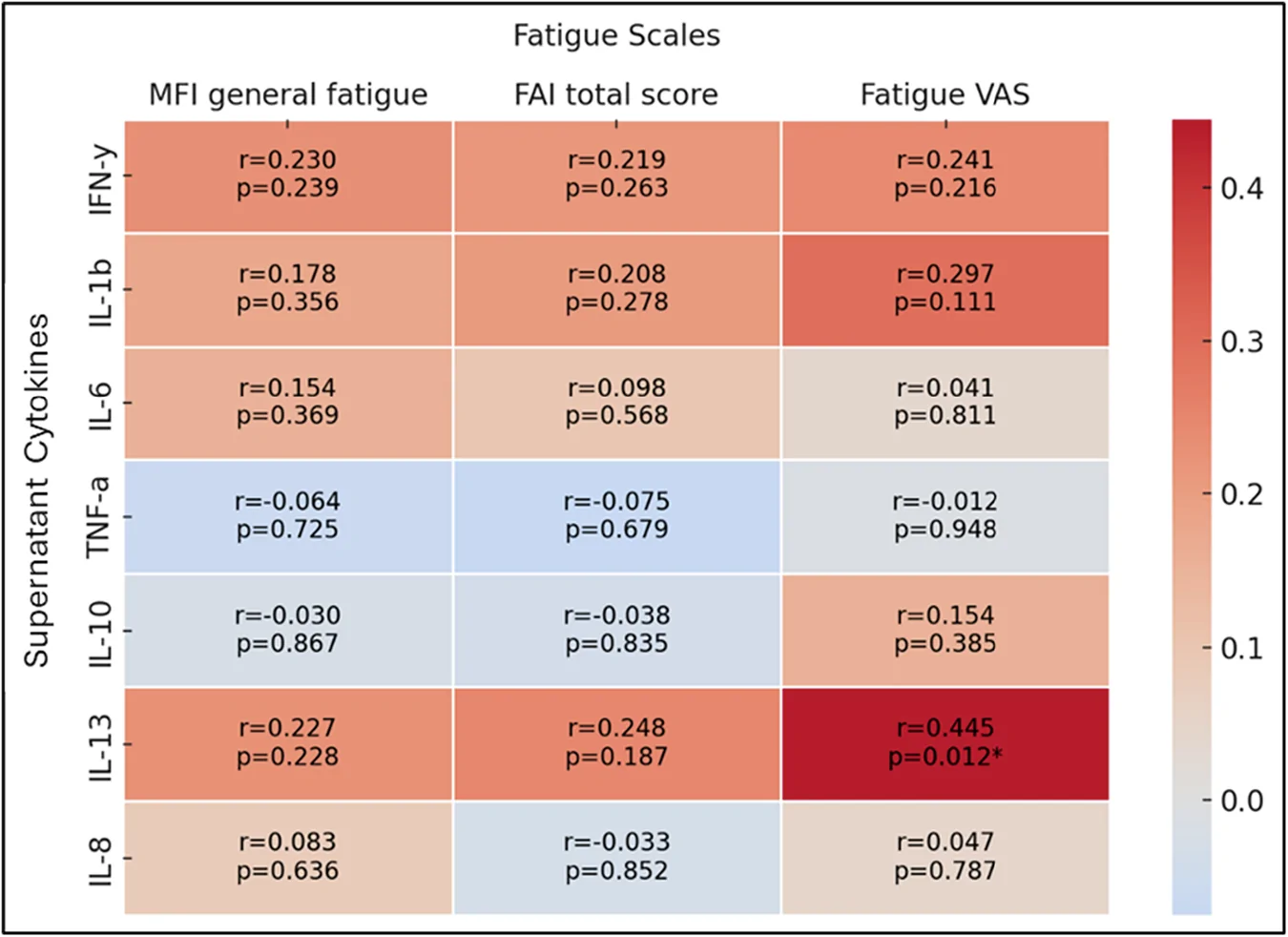
Heatmap showing the correlations between fatigue scales and cytokines in cells’ supernatant. Correlation coefficients (*r*) are displayed inside each cell together with their corresponding *p*-values. The colour scale represents the strength and direction of the correlation, with red indicating positive and blue indicating negative associations. *p* < 0.05 was considered statistically significant and is indicated by an asterisk.

**Fig. 3:**
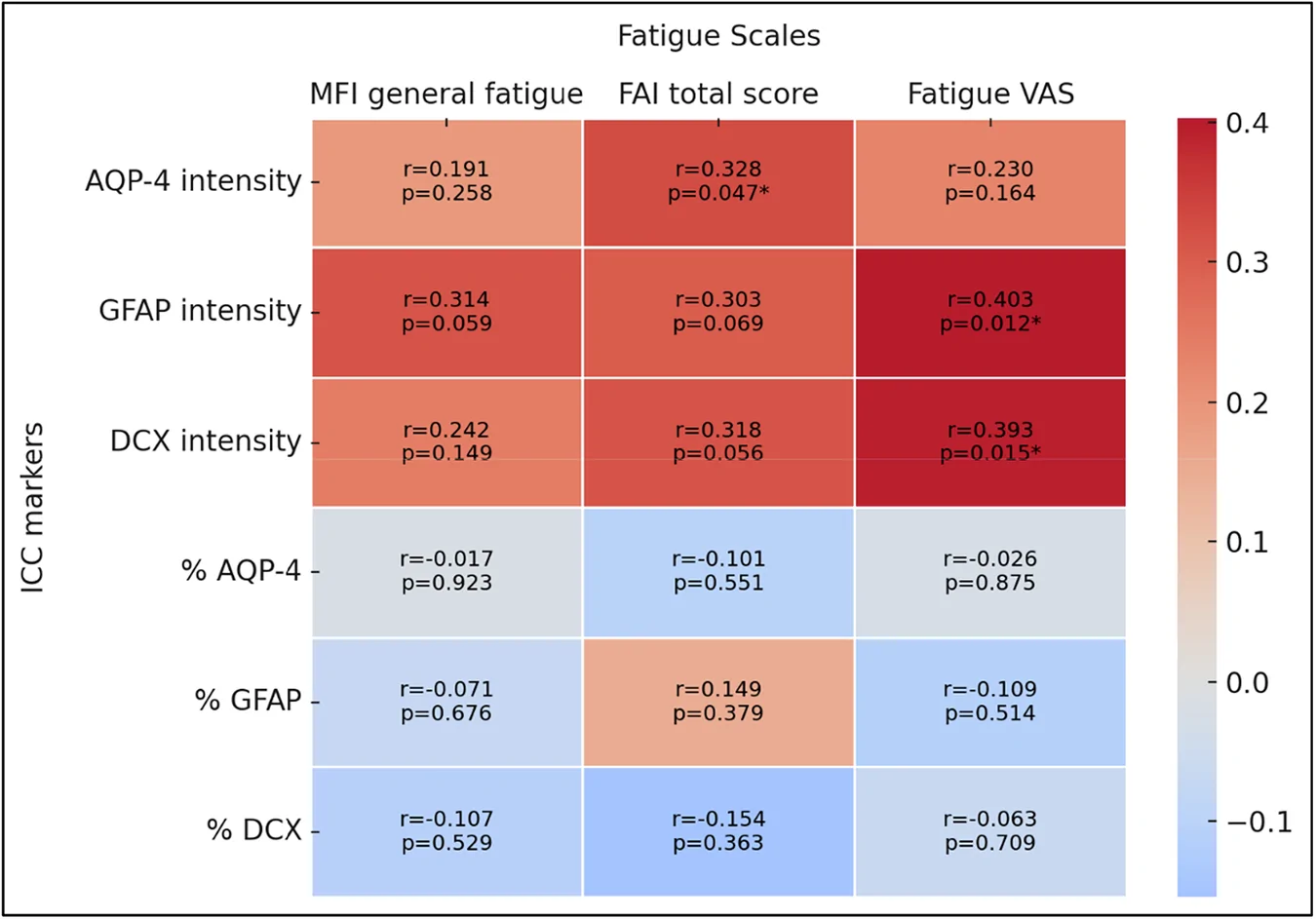
Heatmap showing the correlations between fatigue scales and cytokines. Correlation coefficients (*r*) are displayed inside each cell together with their corresponding *p*-values. The colour scale represents the strength and direction of the correlation, with red indicating positive and blue indicating negative associations. *p* < 0.05 was considered statistically significant and is indicated by an asterisk.

**Fig. 4:**
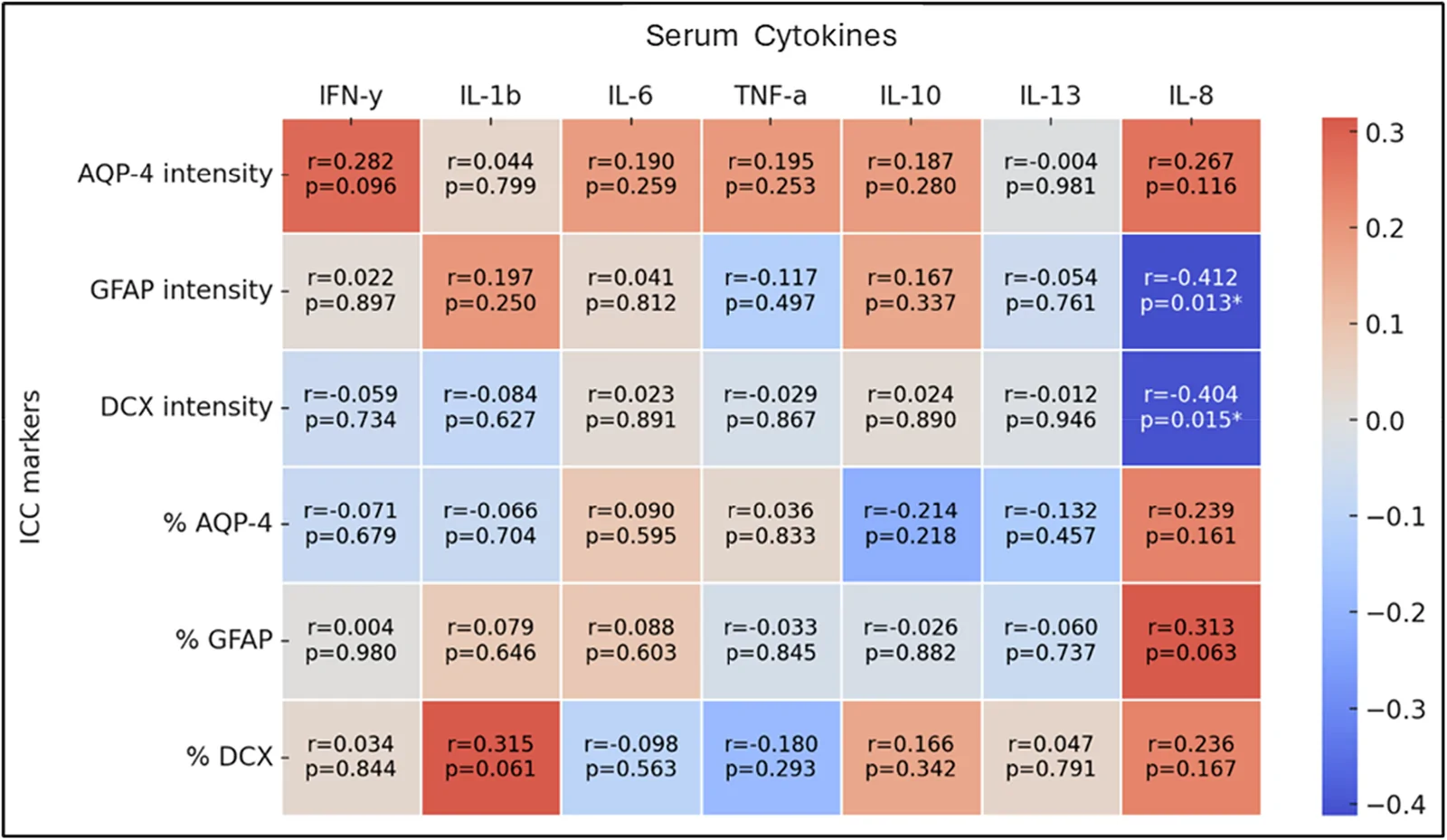
Heatmap showing the correlations between serum cytokines and ICC markers*.* Correlation coefficients (*r*) are displayed inside each cell together with their corresponding *p*-values. The colour scale represents the strength and direction of the correlation, with red indicating positive and blue indicating negative associations. *p* < 0.05 was considered statistically significant and is indicated by an asterisk.

All analyses were performed in R (version 4.3.2; R Core Team, 2025) using RStudio (version 2025.09.0). Before analysis, variables were cleaned and converted to numeric where appropriate, and only complete cases were retained. Pairwise correlations between each clinical, biochemical, immunological and cellular variable were performed. For convenience, results from each correlation analysis were reported either in heatmaps (Figs. 1–4) or in cumulative Tables (Supplementary Tables 1-10). Normality was evaluated using the Shapiro–Wilk test, and either Pearson’s or Spearman’s correlation coefficients were computed accordingly. Given the exploratory nature of the study and the modest sample size, no formal correction for multiple comparisons was applied to avoid excessive Type II error. Results were summarised with correlation coefficients and *p*-values.

**Fig. 5:**
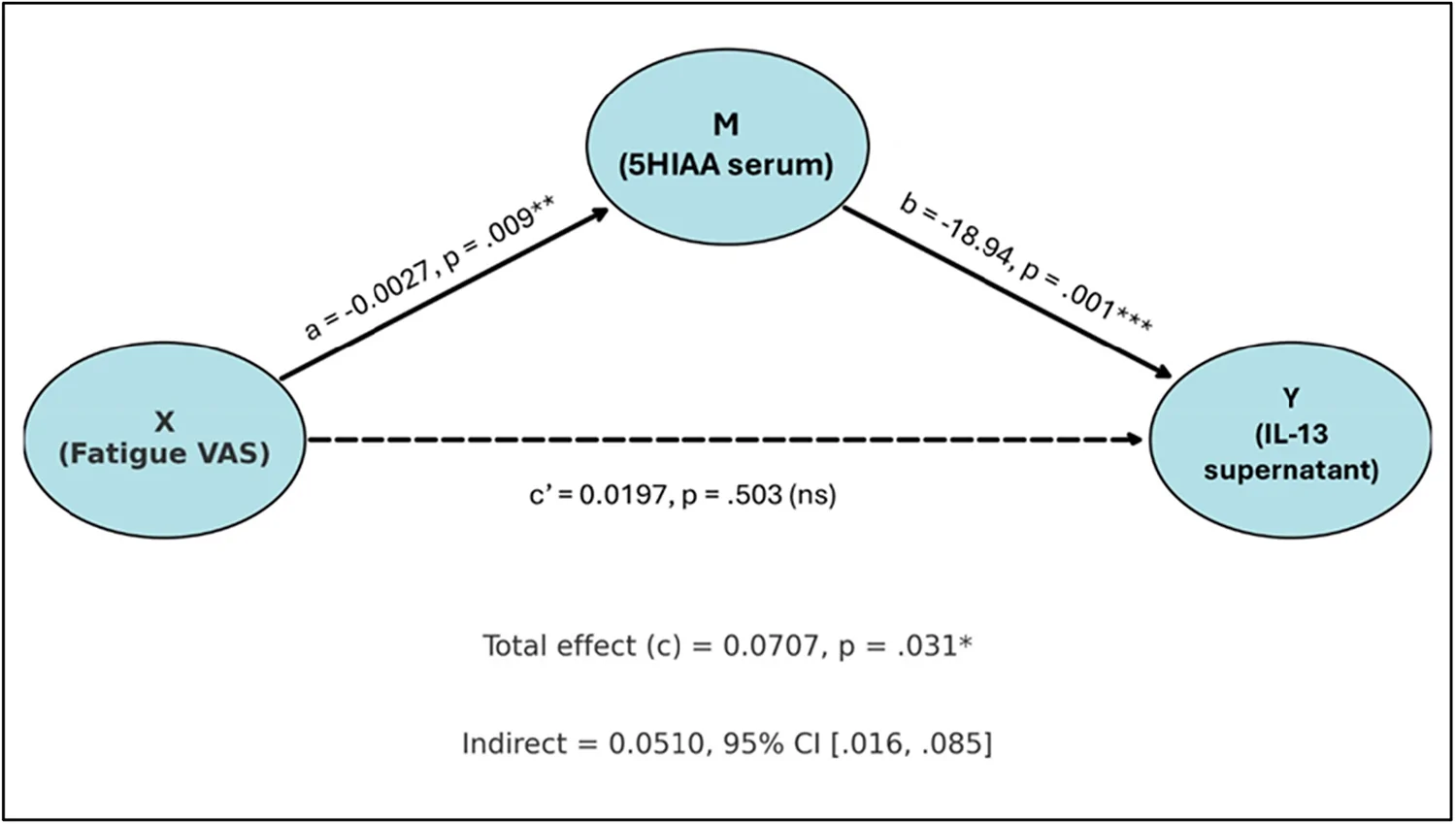
Mediation analysis of the association between fatigue VAS and IL-13 supernatant levels via serum 5HIAA. Fatigue VAS scores (X) were indirectly associated with IL-13 levels (Y) through serum 5HIAA (M). Path a (X - M) was significant (*p*-value: 0.009), as was path b (M - Y, *p*-value: 0.001). The direct effect (c’) of fatigue on IL-13 was nonsignificant (*p*-value: 0.503), while the total effect (c) was significant (*p*-value: 0.031). The indirect effect was significant (estimate = 0.0510, 95% CI [0.016, 0.085]). p < 0.05 was considered statistically significant and is indicated by an asterisk.

Mediation analyses were conducted using the mediation package, with linear regression models fitted to estimate the a, b, c′, and c paths. Indirect effects (average causal mediation effect, ACME), direct effects (ADE), and total effects were estimated using nonparametric bootstrapping with 5 000 simulations. Effect sizes, confidence intervals, and significance levels were reported, and graphical diagrams were generated to illustrate path coefficients and mediation effects (Fig. 5 and Table 2; Supplementary Figs. 1–3 and Supplementary Tables 11–13). Mediation analyses were restricted to variables showing relevant prior associations across fatigue measures, serum biomarkers, and cellular outcomes, rather than applied to all possible marker combinations.

## Results

### Fatigue and serum biomarkers

#### Higher fatigue is associated with lower levels of IL-8 in serum

We first measured levels of candidate cytokines (IL-1β, IL-2, IL-4, IL-6, IL-8, IL-10, IL-12, IL-13, TNF-α, IFN-γ), known to be modulated by the SARS-CoV-2 virus [26], in serum from individuals with past COVID-19 infection (Fig. 1). We found that MFI (r: –0.436, *p*-value: 0.009), FAI (r: –0.407, *p*-value: 0.015), and VAS fatigue scores (r: –0.334, *p*-value: 0.047) were negatively correlated with IL-8 levels in serum. Additionally, there was a trend for a negative correlation between MFI fatigue and TNF-α (r: –0.329, *p*-value: 0.053).

No other cytokines showed significant associations with fatigue measures.

#### Higher fatigue is associated with lower levels of kynurenine pathway and tryptophan-derived and monoamine-related metabolites

We subsequently measured candidate metabolites of the kynurenine pathway and of tryptophan-derived and monoamine-related metabolites in serum from individuals with past COVID-19 infection (Supplementary Tables 1 and 2).

Among kynurenine pathway metabolites, MFI general fatigue scores were significantly negatively correlated with QUIN (r: −0.347, *p*-value: 0.041) and KYN levels (r: −0.358, *p*-value: 0.032). We also observed a negative correlation between Fatigue VAS scores and KYN levels (r: −0.359, *p*-value: 0.029). No other kynurenine pathway metabolites were significantly associated with these fatigue measures.

Regarding monoamine-related metabolites, MFI general fatigue scores were also negatively correlated with the serotonin metabolite 5HIAA (r: −0.395, *p*-value: 0.019). Fatigue VAS scores were negatively correlated with 5HIAA (r: −0.357, *p*-value: 0.033). No other neurotransmitter-related metabolites showed significant associations.

Finally, the FAI total score was not significantly correlated with any metabolite from either pathway

Together, these findings indicate that the most consistent peripheral associations with fatigue involved lower serum IL-8, KYN, and 5-HIAA levels.

### Effects of fatigue on hippocampal cells

Cells were exposed to 1% serum from each individual with past COVID-19 infection, and levels of cytokines, kynurenine pathway and tryptophan-derived and monoamine-related metabolites in the supernatants, as well as imaging markers of astrocytic reactivity and neurogenesis, were quantified.

#### Higher fatigue is associated with higher levels of IL-13 and the kynurenine metabolite ANA in cell supernatant

We then measured the same panel of cytokines, previously quantified in serum, in the supernatant of cells treated with the same serum samples from individuals with past COVID-19 infection (Fig. 2). A significant positive correlation was observed between fatigue VAS scores and IL-13 levels in cell supernatant (r: 0.445, *p*-value: 0.012). No other cytokines showed significant associations with fatigue measures.

Analyses of kynurenine pathway and tryptophan-derived and monoamine-related metabolites in cell culture supernatants (Supplementary Tables 3 and 4) revealed a positive correlation between MFI (r: 0.470, *p*-value: 0.004), FAI (r: 0.369, *p*-value: 0.029), and VAS fatigue scores (r: 0.436, *p*-value: 0.008) and levels of ANA. No other metabolites showed significant associations with fatigue measures.

#### Higher fatigue is associated with increased neurogenesis but also astrocytic reactivity

Finally, we also found a positive correlation between fatigue VAS scores and increased neurogenesis (DCX intensity, r: 0.393, *p*-value: 0.015) and astrocytic reactivity (GFAP intensity, r: 0.403, *p*-value: 0.012), and a positive association between FAI total score and again astrocytic reactivity (AQP4 intensity, r: 0.328, *p*-value: 0.047) (Fig. 3). We also found a trend for a positive correlation between MFI general fatigue score and GFAP intensity (r: 0.314, *p*-value: 0.059), and between FAI total score and DCX intensity (r: 0.318, *p*-value: 0.056) (Fig. 3). Of note, these findings were specific to an increase in the intensity of these cellular outcomes, rather than in the percentage of cells expressing these markers (Fig. 3).

Overall, the most prominent cellular associations with fatigue involved increased IL-13 and ANA release, together with higher DCX and GFAP intensity. While astrogliosis can be the result of such a hyperactive immune response in cells, the increase in neurogenesis could be the result of a possible compensatory mechanism being activated in response to the observed increased neuroinflammation.

### Effects of serum biomarkers levels on hippocampal cells

#### Higher levels of serum cytokines are associated with increased neurogenesis and astrocytic reactivity and decreased production of the kynurenine metabolites in cell supernatant

We subsequently analysed how the levels of cytokines present in the serum of the same individuals with past COVID-19 infection affect hippocampal cells.

Specifically, we found a negative correlation between serum levels of IL-8 and DCX intensity (r: −0.404, *p*-value: 0.0146) and GFAP intensity (r: −0.412, *p*-value: 0.0125), while no other cytokines showed significant associations with these cellular biomarkers. Again, these findings were specific to an increase in the intensity of these cellular outcomes, rather than in the percentage of cells expressing these markers (Fig. 4).

We also found that serum levels of TNF-α negatively correlated with the production of the kynurenine metabolites ANA (r: −0.401, *p*-value: 0.0186), KYN (r: −0.397, *p*-value: 0.0227) and KYNA (r: −0.384, *p*-value: 0.0275) (Supplementary Tables 5 and 6). No other cytokines showed significant associations with any of the metabolites measured.

#### Higher serum levels of kynurenine and serotonin metabolites are associated with lower IL-13 in cell supernatant and higher astrocytic reactivity

Ultimately, we analysed how levels of kynurenine pathway and tryptophan-derived and monoamine-related metabolites in the serum of the same individuals with past COVID-19 infection affect hippocampal cells.

When examining the association between serum kynurenine pathway and tryptophan-derived and monoamine-related metabolites and cytokine levels in cell supernatants (Supplementary Tables 7 and 8), ANA levels were negatively correlated with all measured cytokines, while XANA (r: −0.390, *p*-value: 0.0365) was negatively correlated with IL-13. For serotonin-related metabolites, 5HIAA showed consistent negative correlations across all measured cytokines. Whereas GLU positively correlated only with IL-8 (r: 0.381, *p*-value: 0.0262). No other kynurenine pathway and tryptophan-derived and monoamine-related metabolites displayed significant associations with cytokine levels.

Moreover, correlations between serum kynurenine pathway metabolite levels and hippocampal cell imaging markers (Supplementary Table 9) showed a significant negative correlation between levels of XANA and the percentage of AQP4-positive cells (r: −0.334, *p*-value: 0.046). No other kynurenine pathway metabolites were significantly associated with AQP4, GFAP, or DCX. Among monoamine-related metabolites (Supplementary Table 10), levels of 5HIAA were positively correlated with GFAP intensity (r: 0.347, *p*-value: 0.038). No other neurotransmitter-related metabolites were significantly associated with AQP4, GFAP, or DCX.

These analyses highlighted serum IL-8 and 5-HIAA as peripheral markers associated with downstream hippocampal cell responses.

### Investigation of mediators linking fatigue to cellular outcomes

Finally, we examined whether the effects of fatigue on the cellular outcomes are mediated by cytokines, or by the kynurenine pathway or tryptophan-derived and monoamine-related metabolites produced in serum.

Since there was an association between higher VAS scores and lower serum IL-8 levels, and between lower serum IL-8 and higher neurogenesis (DCX intensity) and astrocytic reactivity (GFAP intensity), we performed a mediation analysis with these three variables. Results show that the direct effect (ADE) of fatigue VAS, independent of IL-8, remain statistically significant on DCX intensity (Estimate = 87.66, 95% CI [27.54, 144.80], *p*-value: 0.007) and GFAP intensity (Estimate = 54.75, 95% CI [14.90, 101.51], *p*-value: 0.009), suggesting that mechanisms beyond IL-8 likely contribute to the relationship between fatigue and both neuronal and astrocytic activation (Supplementary Fig. 1 and Supplementary Table 11; Supplementary Fig. 2 and Supplementary Table 12).

Previous findings also showed an association between higher VAS scores and lower serum 5HIAA levels, and between lower serum 5HIAA and higher supernatant IL-13 levels and increased astrocytic reactivity (GFAP intensity) in the cells. Interestingly, mediation analyses showed that the direct effect (ADE) of fatigue, measured by Fatigue VAS, on IL-13, after accounting for serum 5HIAA, was not statistically significant (Estimate = 0.020, 95% CI [ − 0.040, 0.079], *p*-value: 0.375), indicating that the effect of fatigue on IL-13 is largely explained by 5HIAA mediation. In fact, approximately 71% of the total effect of Fatigue VAS on IL-13 in the supernatant was mediated by 5HIAA, which was statistically significant (*p*-value: 0.036) (Fig. 5, Table 2). These findings suggest that higher fatigue levels may influence IL-13 concentrations predominantly through alterations in the serotonin metabolite 5HIAA.

**Table 2:** Mediation analysis of the association between fatigue VAS and IL-13 supernatant levels via serum 5HIAA.

Effect	Estimate	95% CI Lower	95% CI Upper	*p*-value	Significance
ACME (Indirect Effect)	0.0512	0.0150	0.0873	0.0096	***Significant***
ADE (Direct Effect)	0.0199	−0.0297	0.0682	0.3800	Not significant
Total Effect	0.0711	0.0084	0.1294	0.0232	***Significant***
Proportion Mediated	0.7200	0.2868	2.4858	0.0264	***Significant***

*ACME* average causal mediation effect (indirect effect through serum 5HIAA), *ADE* average direct effect, *Proportion Mediated* proportion of total effect mediated by 5HIAA.

However, when testing serum 5HIAA as a mediator of the relationship between fatigue and GFAP intensity, the direct effect of fatigue remained statistically significant (Estimate = 82.67, *p*-value: < 0.001), while the indirect effect through 5HIAA was not significant (Estimate = −23.94, 95% CI [ − 60.98, 18.54], *p*-value: 0.219). This indicates that 5HIAA does not mediate this association. In fact, approximately 41% of the total effect of fatigue on GFAP intensity was estimated to be mediated by 5HIAA, although this proportion did not reach statistical significance (*p*-value: 0.23) (Supplementary Fig. 3 and Supplementary Table 13).

Overall, these findings suggest that fatigue is associated with neuroimmune and metabolic changes in hippocampal cells, involving serum 5-HIAA and IL-13 release.

## Discussion

This study demonstrates that fatigue following SARS-CoV-2 infection is associated with specific changes in serum cytokines, as well as in the kynurenine pathway and tryptophan-derived and monoamine-related metabolites, which are associated with changes in the function of hippocampal progenitor cells.

Specifically, we observed that: (1) higher fatigue correlated with lower serum IL-8 and kynurenine pathway and tryptophan-derived and monoamine-related metabolites levels; (2) serum from individuals with higher fatigue was associated with increased IL-13 and ANA production in hippocampal cells; (3) fatigue severity was associated with increased immature neuronal (DCX) and astrocytic (GFAP, AQP4) marker expression; (4) serum IL-8 and kynurenine pathway and tryptophan-derived and monoamine-related metabolites were associated with hippocampal cellular outcomes; and (5) mediation analyses revealed that 5HIAA significantly explained the relationship between fatigue and IL-13 release, whereas fatigue effects on GFAP and DCX remained independent of IL-8.

Overall, these findings suggest that fatigue after COVID-19 infection is associated with dysregulation of inflammatory cytokines and the metabolism of the kynurenine pathway and tryptophan-derived and monoamine-related metabolites, and with altered hippocampal neurogenesis-related readouts.

### Fatigue and serum biomarkers

We found that higher fatigue was associated with lower serum IL-8 and the kynurenine pathway, including KYN and QUIN. These findings build on previous work implicating altered tryptophan metabolism following SARS-CoV-2 infection, with reduced tryptophan availability and an increased KYN/TRP ratio [11].

However, our negative correlations between metabolite levels and fatigue severity appear to contrast with this literature. One possible explanation is that immune–metabolic trajectories following COVID-19 infection are dynamic and heterogeneous. Kynurenine pathway activation may be most prominent during acute or subacute inflammatory phases, whereas later stages may be characterised by metabolic depletion, altered pathway regulation, or immune exhaustion, particularly in individuals with ongoing fatigue [6, 27]. Another explanation is compartmentalisation: reductions in circulating metabolites could coincide with increased central nervous system uptake or altered transport across the blood–brain barrier, as suggested in other neuroinflammatory conditions [28]. Future longitudinal studies will be needed to determine whether reduced serum KYN and QUIN reflect depletion, redistribution, or altered central-peripheral balance in fatigue following COVID-19 infection.

The reduction of IL-8 in fatigued individuals is also noteworthy. While much of the post-COVID literature has focused on cytokines such as IL-6, TNF-α, and interferon-γ [4],IL-8 has received comparatively little attention. Although IL-8 is classically known as a neutrophil chemokine, it also influences glial signalling and neurovascular regulation [29]. Our results suggest IL-8 could be a key, yet underexplored, biomarker for fatigue, specifically following SARS-CoV-2 infection.

### Fatigue and hippocampal cellular outcomes

When hippocampal progenitors were treated with serum from individuals with past COVID-19 infection, we observed an association between fatigue and higher IL-13 and ANA release in cell supernatant, alongside higher GFAP, AQP4, and DCX intensity. These findings indicate that serum from individuals with higher fatigue was associated with changes in hippocampal differentiation-related markers, astrocytic activation, and neurogenesis-related processes in vitro.

Although these endpoints are intermediate cellular markers rather than direct measures of behavioural or cognitive function, they provide biologically meaningful readouts of hippocampal cell responses [24, 25]. Given that the extent of adult hippocampal neurogenesis in humans remains uncertain, DCX should be interpreted here as a neurogenesis-related marker within the in vitro model, rather than as direct evidence of neurogenesis in vivo. Together, the imaging, cytokine, and metabolite data suggest altered neuroimmune and metabolic activity within the culture system.

In this study, we observed an increased fluorescence intensity (astrogliosis) rather than a higher percentage of marker-positive cells (astrogliogenesis), suggesting that fatigue-associated serum protein expression is neuroinflammatory in nature and does not affect cell fate.

While increased DCX intensity is consistent with enhanced neurogenesis-related activity in this model, it may also reflect changes in neuronal maturation or stress-related cellular responses. Similarly, increased GFAP and AQP4 expression may represent astrocytic responses involved in maintaining homeostasis, although their interpretation may depend on context and duration.

In particular, the concurrent rise in GFAP and AQP4 suggests astrocytic activation, although interpretation requires caution. GFAP expression may indicate compensatory or reactive responses depending on the inflammatory context. Similarly, AQP4 upregulation can be adaptive, supporting neurovascular coupling and metabolic balance, or pathological when sustained, reflecting reactive gliosis and disrupted glymphatic function [30, 31]. Our results likely capture a shift toward a reactive astrocytic phenotype that may initially aid homeostasis but become detrimental if persistent.

Interestingly, the parallel increase in DCX intensity could represent a compensatory neurogenic response to this inflammatory environment. Cytokine and kynurenine pathway activation may trigger astrocytic reactivity while simultaneously stimulating progenitor proliferation to maintain or restore hippocampal function. This combination of inflammatory activation and compensatory neurogenesis has also been noted in other neuroimmune contexts, where an initially adaptive response may become maladaptive under chronic immune stress [19, 25].

Finally, the rise in IL-13 may represent an anti-inflammatory compensatory response, given the role of IL-13 in modulating glial reactivity and promoting repair [32], though persistent IL-13 elevation could also contribute to maladaptive immune or metabolic regulation [33]. Likewise, elevated ANA suggests metabolic–immune crosstalk influencing cell behaviour [18].

Overall, these findings provide evidence that fatigue after COVID-19 infection is linked to immune-mediated alterations in hippocampal cell function, affecting both glial and neuronal compartments. This is, to our knowledge, the first evidence that serum from individuals with past COVID-19 infection is associated with changes in human hippocampal neuronal and astrocytic markers in vitro, offering mechanistic insight into how peripheral immune signals may contribute to neurocognitive and affective symptoms in these individuals. Evidence from translational neuropsychiatric studies further supports the notion that COVID-19 infection can have downstream relevance for brain biology, providing a broader context for experimental approaches examining post-infectious immune–brain interactions [34].

### Effects of serum cytokines and kynurenine pathway and tryptophan-derived and monoamine-related metabolites on hippocampal cells

We found that serum IL-8 was inversely correlated with GFAP and DCX expression, while TNF-α showed negative associations with ANA, KYN, and KYNA in culture supernatants. These findings are consistent with established evidence that inflammatory cytokines, including TNF-α and IL-1β, regulate enzymes of the kynurenine pathway, such as indoleamine 2,3-dioxygenase (IDO), thereby shaping downstream metabolite availability[14, 15]. Our data suggest that changes in cytokine balance in individuals with past COVID-19 infection may contribute to altered metabolic signalling, which in turn affects hippocampal cell function.

The role of IL-8 is particularly interesting. Although best known as a neutrophil chemokine, IL-8 also acts within the central nervous system, influencing astrocyte communication, synaptic activity, and neurovascular coupling [29]. The lower IL-8 levels observed in individuals with higher fatigue after COVID-19 infection could therefore reflect reduced neuroimmune support or an adaptive response following earlier inflammatory activation. Some experimental studies have shown that IL-8 can promote neuronal survival and angiogenesis under stress conditions [35, 36], so its reduction might contribute to a less supportive environment for hippocampal plasticity.

The negative correlations between TNF-α and kynurenine metabolites, including KYNA, may point to a shift from early activation to later metabolic exhaustion within the kynurenine pathway. While TNF-α can initially promote tryptophan degradation through IDO induction, sustained immune activation may suppress the pathway or deplete substrate availability over time[16]. Such dynamics could explain why we observed lower kynurenine metabolites despite ongoing immune dysregulation. Since KYNA and ANA play neuroprotective and antioxidant roles, reduced levels might further compromise cellular homeostasis and differentiation [28].

Overall, these findings point to a close interplay between cytokine and metabolic networks in individuals with past COVID-19 infection. By linking participants’ serum immune-metabolic profiles to changes in hippocampal progenitor behaviour, this study provides direct evidence that COVID-19-related immune alterations can influence cellular processes relevant to neuroplasticity and fatigue.

### Investigation of mediators linking fatigue to cellular outcomes

Mediation analysis indicated that serum 5HIAA significantly explained the relationship between fatigue and IL-13 release in vitro, while the effects of fatigue on GFAP and DCX were not mediated by IL-8. 5HIAA is the primary metabolite of serotonin, produced via monoamine oxidase–dependent degradation, and is widely used as an indirect marker of serotonin turnover. Altered serotonin metabolism has been linked to mood changes, fatigue, and immune dysregulation in inflammatory conditions [9]. In parallel, disturbances in tryptophan metabolism influence the balance between the serotonin and kynurenine pathways, shifting substrate away from serotonin synthesis toward kynurenine production under inflammatory states [37, 38]. The finding that 5HIAA mediated the association between fatigue and IL-13 release, therefore, suggests that disrupted serotonin turnover may interact with cytokine pathways after COVID-19 infection, suggesting a possible link between altered monoamine metabolism, immune activation, and fatigue.

### Limitations and future directions

While this study included a modest cohort and used an in vitro model that cannot capture the full cellular complexity of the brain, it provides a useful translational approach to link patient immune-metabolic profiles with hippocampal cell biology. The model also does not reproduce the selective properties of the blood–brain barrier. However, recent studies reporting blood–brain barrier disruption in acute COVID-19 and long COVID-associated cognitive impairment support the broader relevance of peripheral-to-brain signalling after SARS-CoV-2 infection [39, 40].

The absence of comparator groups, such as uninfected individuals or post-COVID individuals without fatigue, limits the ability to determine whether these findings are specific to SARS-CoV-2 infection. However, our aim was to examine associations within a biologically confirmed post-COVID cohort using fatigue severity as a continuous measure, rather than to establish disease specificity.

These findings should be interpreted with caution, given the number of statistical tests performed and the associated risk of inflated false positive rates. Although several associations were significant, the observed correlations were generally modest, corresponding to approximately 9–16% of shared variance for several associations, and likely capture only part of the biological complexity underlying fatigue. Similarly, although the mediation analysis suggested that 5-HIAA may partly explain the association between fatigue and IL-13 release, this result should be considered preliminary and hypothesis-generating given the modest sample size. In addition, metabolite levels measured in cell supernatants reflect the combined contribution of baseline media composition, cellular uptake, and cellular release, and should therefore be interpreted as net supernatant levels.

Finally, time since first infection was positively associated with fatigue severity across all measures, suggesting some temporal heterogeneity in fatigue presentation across the post-infection period. Vaccination status was also not systematically controlled for and may have contributed to variability in immune and metabolic profiles. Future work should aim to replicate these findings in larger, longitudinal cohorts with appropriate comparator groups and to extend the cellular model to include additional brain-relevant cell types, such as microglia.

## Conclusion

Overall, our findings indicate that fatigue after COVID-19 infection is associated with neuroimmune and metabolic changes in hippocampal cells, involving peripheral serotonin metabolism (5-HIAA) and cytokine signalling (IL-13).To our knowledge, this is the first study to integrate patient serum profiling with human hippocampal cell assays to demonstrate how immune–metabolic disturbances following COVID-19 infection translate into impaired neuroplasticity. Collectively, these results identify potential biomarkers and therapeutic targets, providing novel mechanistic insight into the biological basis of post-COVID-19 fatigue.

## Supplementary information

Supplementary material

## Data Availability

All data will be available upon request.
