# Supplementary material for: Fatigue after COVID-19 infection is associated with peripheral immunometabolic alterations affecting neuroimmune responses in the hippocampus
Source: Transl Psychiatry. 2026 Jul 15;16:543. doi: 10.1038/s41398-026-04250-9 (PMC13650122; doi:10.1038/s41398-026-04250-9)
Supplement: Supplementary file 1 [file 41398_2026_4250_MOESM1_ESM.docx]

**Supplementary Table 1:** *Correlations between fatigue scales and KYN metabolites in serum*

**Supplementary Table 2:** *Correlations between fatigue scales and tryptophan-derived and monoamine-related metabolites in serum*

**Supplementary Table 3:** *Correlations between fatigue scales and KYN pathway metabolites in cells’ supernatant*

**Supplementary Table 4:** *Correlations between fatigue scales and tryptophan-derived and monoamine-related metabolites in cells’ supernatant*

**Supplementary Table 5:** *Correlation between cytokines in serum and KYN pathway metabolites in cells’ supernatant*

**Supplementary Table 6:** *Correlation between cytokines in serum and tryptophan-derived and monoamine-related metabolites in cells’ supernatant*

**Supplementary Table 7:** *Correlation between KYN pathway metabolites in serum and cytokines in cells’ supernatant*

**Supplementary Table 8:** *Correlation between tryptophan-derived and monoamine-related metabolites in serum and cytokines in cells’ supernatant*

**Supplementary Table 9:** *Correlation between KYN pathway metabolites in serum and ICC markers*

**Supplementary Table 10:** *Correlation between tryptophan-derived and monoamine-related metabolites in serum and ICC markers*

**Supplementary Figure 1:** *Mediation analysis of the association between fatigue VAS and DCX intensity via IL-8.*

Fatigue VAS scores (X) were not significantly associated with IL-8 levels (M; path *a*, *p* = 0.276). The path from IL-8 to DCX intensity (Y) showed a trend toward significance (path *b*, *p* = 0.051). The direct effect (*c’*) of fatigue on DCX was significant (*p* = 0.008), and the total effect (*c*) was also significant (*p* = 0.004). The indirect effect (ACME) was not significant (estimate = 12.0, 95% CI [–9.4, 46.1], *p* = 0.277).

p < 0.05 was considered statistically significant and is indicated by an asterisk.

**Supplementary Table 11:** *Mediation analysis of the association between fatigue VAS and DCX intensity via IL-8.* ACME = Average Causal Mediation Effect (indirect effect through serum IL-8), ADE = Average Direct Effect, Proportion Mediated = Proportion of total effect mediated by IL-8.

| **Effect** | **Estimate** | **95% CI Lower** | **95% CI Upper** | **p-value** | **Significance** |
| --- | --- | --- | --- | --- | --- |
| **ACME (Indirect Effect)** | 12.01 | -9.41 | 46.10 | 0.277 | Not significant |
| **ADE (Direct Effect)** | 87.66 | 27.54 | 144.80 | 0.0068 | **Significant** |
| **Total Effect** | 99.68 | 38.81 | 158.37 | <0.001 | ***Significant*** |
| **Proportion Mediated** | 0.12 | -0.13 | 0.50 | 0.277 | Not significant |

**Supplementary Figure 2:** *Mediation analysis of the association between fatigue VAS and GFAP intensity via IL-8.*

Fatigue VAS scores (X) were not significantly associated with IL-8 levels (M; path a, p = 0.276). The path from IL-8 to GFAP intensity (Y) was non-significant (path b, p = 0.120). The direct effect (c’) of fatigue on GFAP showed a trend (p = 0.076), while the total effect (c) was significant (p = 0.041). The indirect effect (ACME) was not significant (estimate = 9.0, 95% CI [–9.0, 27.7], p = 0.308).

p < 0.05 was considered statistically significant and is indicated by an asterisk.

**Supplementary Table 12:** *Mediation analysis of the association between fatigue VAS and GFAP intensity via IL-8.* ACME = Average Causal Mediation Effect (indirect effect through serum IL-8), ADE = Average Direct Effect, Proportion Mediated = Proportion of total effect mediated by IL-8.

| **Effect** | **Estimate** | **95% CI Lower** | **95% CI Upper** | **p-value** | **Significance** |
| --- | --- | --- | --- | --- | --- |
| **ACME (Indirect Effect)** | 9.011 | -9.020 | 27.749 | 0.3084 | Not significant |
| **ADE (Direct Effect)** | 54.754 | 14.905 | 101.505 | 0.0092 | **Significant** |
| **Total Effect** | 63.765 | 23.816 | 108.525 | 0.0004 | ***Significant*** |
| **Proportion Mediated** | 0.141 | -0.187 | 0.523 | 0.3080 | Not significant |

**Supplementary Figure 3:** *Mediation analysis of the association between fatigue VAS and GFAP intensity via 5HIAA.*

Fatigue VAS scores (X) were not significantly associated with 5HIAA serum levels (M; path a, p = 0.349). The path from 5HIAA serum to GFAP intensity (Y) was highly significant (path b, p < 0.001. The direct effect (c’) of fatigue on GFAP remained significant (p < 0.001), whereas the total effect (c) showed a statistical trend (p = 0.068). The indirect effect (ACME) was not significant (estimate = −23.94, 95% CI [−60.98, 18.54], p = 0.219).

p < 0.05 was considered statistically significant and is indicated by an asterisk.

**Supplementary Table 13:** *Mediation analysis of the association between fatigue VAS and GFAP intensity via 5HIAA.* ACME = Average Causal Mediation Effect (indirect effect through serum *5HIAA*), ADE = Average Direct Effect, Proportion Mediated = Proportion of total effect mediated by *5HIAA*.

| **Effect** | **Estimate** | **95% CI Lower** | **95% CI Upper** | **p-value** | **Significance** |
| --- | --- | --- | --- | --- | --- |
| **ACME (Indirect Effect)** | -23.94 | −60.98 | 18.54 | 0.219 | Not significant |
| **ADE (Direct Effect)** | 82.67 | 38.60 | 121.32 | <.001 | ***Significant*** |
| **Total Effect** | 63.765 | 23.816 | 108.525 | 0.008 | ***Significant*** |
| **Prop. Mediated** | −0.41 | −2.28 | 0.230 | 0.226 | Not significant |
